# Co-occurrence of Aggression and Suicide Attempt Among Young People and Related Factors: Findings from Iranian Youth Cohort Study in Ravansar

**DOI:** 10.34172/aim.2023.49

**Published:** 2023-06-01

**Authors:** Habibolah Khazaie, Behrooz Hamzeh, Farid Najafi, Azita Chehri, Afarin Rahimi-Movaghar, Masoumeh Amin-Esmaeili, Mehdi Moradi-Nazar, Ali Zakiei, Yahya Pasdar

**Affiliations:** ^1^Sleep Disorders Research Center, Kermanshah University of Medical Sciences, Kermanshah, Iran; ^2^Research Center for Environmental Determinants of Health, School of Public Health, Kermanshah University of Medical Sciences, Kermanshah, Iran; ^3^Social Development and Health Promotion Research Center, Kermanshah University of Medical Sciences, Kermanshah, Iran; ^4^Department of Psychology, Kermanshah Branch, Islamic Azad University, Kermanshah, Iran; ^5^Iranian National Center for Addiction Studies (INCAS), Tehran University of Medical Sciences, Tehran, Iran; ^6^Department of Epidemiology, Kermanshah University of Medical Sciences, Kermanshah, Iran Received: September

**Keywords:** Aggression, Mental disorder, Suicide, Youth

## Abstract

**Background:**

Aggression and suicide attempt are behaviors that affect public health. To better understand the nature of these behaviors, the present study was conducted to investigate the concurrence of suicide attempt and aggression in young people. This study sought to identify those factors which contribute to the co-occurrence of suicide attempt and aggression in young people.

**Methods:**

The present study was part of the recruitment phase of Ravansar youth cohort study (a branch of the PERSIAN Cohort) with a sample of 2991 people from Ravansar in western Iran. Registration and data collection were done between October 2014 and January 2017. Data were collected using structured interviews and standard questionnaires, and analyzed using multi-nominal logistic regression analysis.

**Results:**

The results showed that the variables of age and education were significant correlates of concurrence of suicide attempt and aggression. Also, among the mental disorders, only major depressive disorder (MDD) (OR=8.34, *P*=0.001) predicted the concurrence of suicide attempt and aggression in the past 12 month. In contrast, the variables of generalized anxiety disorder (GAD), obsessive-compulsive disorder (OCD), dysthymia, and substance use disorder were not significant in predicting the concurrence of attempted suicide and aggression.

**Conclusion:**

The occurrence of aggression and suicide in young people is a complex phenomenon, and more research should be done to better understand this phenomenon.

## Introduction

 Young people are the human capital of any society; so, addressing their mental health is a public health priority. Suicidal and aggressive behaviors are among the issues that endanger the health of young people. Aggression is a common and costly behavior.^1^ Research indicates a significant prevalence of aggressive behaviors in the young population.^2,3^ On the other hand, aggression can be a real risk.^4^ which can have dangerous consequences,^5^ such as causing self-harm, suicide, harming others, or homicide.^6^ In the literature, aggression is often defined as behavior that is intended to harm another person.^7^

 Aggression is associated with a variety of factors, including individual, family, and environmental factors.^8^ substance use disorder, alcohol drinking, gender, and unemployment have also been reported to predict aggression.^9^ The results of a study also showed that there is an association between aggression and depression.^10^ Other research has shown higher level of anxiety as a risk factor for aggression.^11^

 Suicide attempt was another dependent variable investigated in this study. Mortality rates due to suicide are significant in different parts of the world and suicide is one of the leading causes of death worldwide, especially in youths.^12^ Suicide is attempted in a variety of ways, and varies according to demographic variables such as gender and age.^13,14^ On the other hand, suicide attempt is a global phenomenon that is seen in all parts of the world, and in Middle Eastern countries including Iran, it is a threat to public health. The results of a study showed that in Middle Eastern countries, factors such as drug use and alcohol drinking are associated with suicide attempt.^15^

 Suicide is associated with psychiatric disorders, and research has shown that depression, anxiety, and substance use disorder are the most important risk factors for suicide.^16^ The results of a study showed that a wide range of mental disorders predict the likelihood of experiencing suicidal ideation, but anxiety is the most important factor that can predict suicide attempt in people with suicidal ideation.^17^ The results of another study showed that most people who attempted suicide had depressive disorder.^12^ As far as we know, many studies have been done on suicidality and the results are sometimes contradictory. Therefore, it is necessary to conduct more studies to determine the reasons for the contradictions. On the other hand, this can be an interesting challenge to investigate the mechanism by which suicidal ideation is translated into action in individuals. Some researchers have suggested that more smart and vigorous research is needed to understand the mechanism of the association between mental disorders and suicide.^18^

 A body of studies have examined aggression and suicide alone, but less research has been done on the co-occurrence of these two behaviors. It has been noted before that the occurrence of either of these behaviors alone has serious consequences on public health, but if these two occur simultaneously, what aspects of health will be harmed? One study showed that alcohol drinking and post-traumatic stress disorder were effective in the concurrence of aggression and suicide in veterans.^19^ However, it is necessary to conduct more research in samples such as young people, as collecting information on suicidal and aggressive behaviors can be used to treat and prevent these harmful behaviors.

 Iran has experienced rapid social, economic, and demographic changes in recent decades, and now young people constitute a significant proportion of the total population.^20^ Investigating the impact of these changes on the young population can help with more precise planning to promote mental health, because proper planning requires basic information, epidemiological studies and correct knowledge of the problem.^21^ On the other hand, planning and providing mental health services can reduce mental health problems.^22^ Therefore, the present study was conducted to investigate the concurrence of suicide attempt and aggression in young people. This study sought to determine what factors contribute to the co-occurrence of suicide attempt and aggression in young people, the prevalence of aggression and suicide, and the disorders correlated with aggression and suicide.

## Materials and Methods

###  Design and Background

 The PERSIAN Youth Cohort (PYC) study is being run as a supplement of the main Prospective Epidemiological Research Studies in Iran (PERSIAN) Cohort study. The PERSIAN Cohort is a nationwide cohort study launched in the year 2014 which covers 170 000 adults aged 35-70 years.^23,24^ The study is designed as a research platform to investigate a variety of exposures and outcomes related to physical health such as cardiovascular diseases and provided an infrastructure for implementation of other potential cohort studies. Several sub-cohort studies including the PERSIAN birth cohort, PYC, and elderly cohorts were appended to the main PERSIAN cohort study over the past years (http://persiancohort.com/aboutus/). The PYC mainly investigates incidence, course, and the correlated factors of common psychiatric disorders as well as substance use disorder, suicide attempt, traffic and non-traffic injuries, outpatient and inpatient psychiatric service use, and death.

###  Sample

 The first wave of the youth cohort includes 9000 people from three geographical regions of Iran. The present study focused on data drawn from Ravansar County. The inhabitants of Ravansar are mostly Kurd ethnics with a limited number of immigrant population of other ethnicities which makes it a good candidate to be included in the PYC in which a limited number of Kurdish ethnics have participated. The population of Ravansar County was around 50 000 according to the latest national census conducted in 2016. A total sample of 3000 urban and rural youth individuals aged 15 to 34 who resided in Ravansar for at least 6 months was randomly selected. This sample size was determined using 80% power if alpha equals 0.05 (incidence in non-exposed = 0.03; exposed = 0.02). In addition to the age criterion, people were excluded if they temporarily resided in Ravansar (e.g. due to temporary work assignment or military service), or if they were unable to respond to questions because of acute severe or chronic permanent medical or psychiatric illnesses, or unable to understand the Persian language. However, the young generation in the city was mostly literate and could understand and speak in Persian. Only nine people were excluded due to incomplete interviews.

###  Data Collection

 The registration and data collection of the first phase were completed from April 2015 to April 2017 in the cohort center located in an urban area. A face-to-face interview was conducted by trained clinical psychologists or counselors, after receiving necessary information regarding the study, ensuring privacy and confidentiality of the information, and signing the informed consent. Each interview took an average of 60 minutes. Participants’ responses were recorded in an electronic online questionnaire connected to the central cohort server. The quality of the work process was monitored daily by a general practitioner as field manager. The data were continuously assessed by the data management center located in Tehran University of Medical Sciences for completeness of the information, detecting possible errors, and providing technical feedback.

###  Data Collection Tools

 A structured interview was carried out using Composite International Diagnostic Interview, version 2.1 (based on DSM-IV-TR) to assess the lifetime prevalence of psychiatric disorders including major depressive disorder (MDD), generalized anxiety disorder (GAD), persistent depressive disorder (PDD or dysthymia), obsessive-compulsive disorder (OCD), drug use disorders (opioid and stimulants), and alcohol use disorder.

 The Persian version of lifetime Suicidal Thoughts and Behaviors was adapted from the version used in the Health Mental World (WMH) Survey.^23^ It has been already used in the Iranian Mental Health Survey (IranMHS).^25^ This questionnaire includes 9 items related to suicide, including suicide attempts. Proper inter-rater reliability has been reported for this tool in Iranian samples.^25^ Only a dichotomous question regarding a suicide attempt in the past 12 months was used for the current study.

 Physical aggression was assessed using a questionnaire consisting of six items in two sections which assess the occurrence and seriousness of any violent behavior in the last 12 months. This instrument is reported to have appropriate inter-rater reliability in the general population of Iran with a kappa agreement coefficient of 0.6.^25^ The first part of the questionnaire has four yes/no questions evaluating any form of violent behavior including destruction of properties, self-harm, domestic violence, and violence against people outside the family. Aggression in the past 12 months was defined as a positive answer to any of the four questions.

 Socio-demographic characteristics, consisting of gender, age, number of years of completed education, and marital status, were also collected. In addition, self-reported history of any psychiatric disorders including depressive and anxiety disorders, OCD, bipolar disorders, other severe psychiatric disorders with delusion and hallucination, and addiction were assessed in the first degree family members. Participants were also asked whether the diagnosis was confirmed by a mental health care specialist or medical doctor.

###  Analysis 

 Statistical analysis was performed using the SPSS 20 software (IBM Corp., Armonk, NY, USA). A descriptive analysis was performed and mean and standard deviation for continuous variables and proportions for categorical variables were provided. The chi-square test was used to examine the differences between the groups due to the categorical nature of mental disorders. In the next step, multinomial logistic regression analysis was performed to predict concurrence of suicide attempt and aggression. All socio-demographic variables (gender, age, marital status, education, place of residence) were entered into the model simultaneously. The results were presented as adjusted odds ratios (OR) with 95% confidence intervals (CIs). All tests were 2-tailed and in the analysis, *P* value < 0.05 was considered statistically significant.

## Results

 In this study, the data of 2991 people were analyzed, of whom 1663 (55.6%) were women and 1328 (44.4%) were men. The participants’ age ranged from 15 to 34 years (Median = 28); 313 people (10.5%) were in the age group of 15 to 19 years (Median = 18), 610 people (20.4%) were in the age group of 20 to 24 years (Median = 22), 902 people (30.2%) were in the age group of 25 to 29 years (Median = 27), and 1166 people (39%) were in the age group of 30 to 34 years (Median = 32). High school education was the most frequent level (40.9%) among individuals. More than half of the participants (56%) lived in the city. More details on descriptive information are presented in Table 1.

**Table 1:** Sample Characteristics, Descriptive Statistics and Suicide Prevalence and Aggression Rates

**Variables**		**Total Sample** **N=2991**	**Suicide** **(Life Time)** **N=156 **	**Suicide** **(Past 12 Month)** **N=45**	**Aggression ** **N=1439**	**Concurrent** **N=39**	**Neither Behavior** **N=1519**
Gender	Female	1663 (55.6)	111 (71.2)	30 (66.7)	823 (57.2)	25 (64.1)	813 (53.5)
	Male	1328 (44.4)	45 (28.8)	15 (33.3)	616 (42.8)	14 (35.9)	706 (46.5)
Age	15-19	313 (10.5)	19 (12.2)	11 (24.4)	189 (13.1)	11 (28.2)	122 (8)
	20-24	610 (20.4)	47 (30.1)	15 (33.3)	288 (20)	14 (35.9)	313 (20.6)
	25-29	902 (30.2)	44 (28.2)	11 (24.4)	409 (28.4)	7 (17.9)	478 (31.5)
	30-34	1166 (39)	46 (29.5)	8 (17.8)	553 (38.4)	7 (17.9)	606 (39.9)
Education	Illiterate	11 (4)	0	0	5 (0.3)	0	6 (0.4)
	Primary school	481 (16.1)	35 (22.4)	7 (15.6)	251 (17.4)	6 (15.4)	223 (14.7)
	Middle school	530 (17.7)	28 (17.9)	11 (24.4)	258 (17.9)	9 (23.1)	264 (17.4)
	High school	1222 (40.9)	70 (44.9)	22 (48.9)	632 (43.9)	20 (51.3)	575 (37.9)
	University	747 (25)	23 (14.7)	5 (11.1)	293 (20.4)	4 (10.3)	451 (29.7)
Residence	Urban	1674 (56)	98 (62.8)	28 (62.2)	848 (58.9)	25 (64.1)	806 (53.1)
	Rural	1317 (44)	58 (37.2)	17 (37.8)	591 (41.1)	14 (35.9)	713 (46.9)
Marital status	Never married	1227 (41)	51 (32.7)	22 (48.9)	519 (36.1)	21 (83.8)	699 (46)
	Married	1668 (55.8)	82 (52.6)	18 (40)	869 (60.4)	15 (38.5)	783 (51.5)
	Previously married	96 (3.2)	23 (14.7)	5 (11.1)	51 (3.5)	3 (7.7)	37 (2.4)
Psychiatric disorder	GAD	190 (6.4)	37 (23.7)	7 (15.6)	130 (9)	7 (17.9)	56 (3.7)
	MDD	646 (21.6)	106 (67.9)	31 (68.9)	397 (27.6)	28 (71.8)	230 (15.1)
	Dysthymia	57 (1.9)	5 (3.2)	1 (2.2)	38 (2.6)	1 (2.6)	17 (1.1)
	OCD	226 (7.6)	35 (22.4)	10 (22.2)	150 (10.4)	10 (25.6)	71 (4.7)
	Substance use disorder	152 (5.1)	13 (8.3)	3 (6.7)	109 (7.6)	3 (7.7)	41 (2.7)

Table 1 shows that 156 people had a history of suicide attempt (in life time), of whom 71.2% were women and 28.8% were men; 12.2% were aged 15 to 19 years, 30.1% were aged 20 to 24 years, 28.2% were aged 25 to 29 years, and 29.5% were aged 30 to 34 years. High school education was the most frequent level of education (44.9%) among these people. The majority of people were urban residents (62.8%) and 37.2% lived in rural areas. More than half of the participants (52.6%) were married. Analysis of psychiatric disorders among individuals with a history of suicide attempt showed that 23.7% had GAD, 67.9% had MDD, 3.2% had dysthymia, 22.4% had OCD, and 8.3% had a history of substance abuse.

Table 1 also shows that 45 people had a history of suicide attempt (in the past 12 months), of whom 66.7% were women and 33.3% were men; 33.3% were aged 15 to 19 years, 24.4% were aged 20 to 24 years, 33.3% were aged 25 to 29 years, and 24.4% were aged 30 to 34 years. High school education was the most frequent level of education (48.9%) among these people. The majority of individuals were urban residents (62.2%) and 37.8% lived in rural areas, and 40% were married. Analysis of psychiatric disorders among individuals with a history of suicide attempt in the past 12 months showed that 16.6% had GAD, 68.9% had MDD, 2.2% had dysthymia, 22.2% had OCD, and 6.7% had a history of substance abuse.

 The results also showed that 1439 had a history of aggressive behaviors, of whom 57.2% were women and 42.8% were men, 13.1% were aged 15 to 19 years, 20% were aged 20 to 24 years, 28.4% were aged 25 to 29 years, and 38.4% were aged 30 to 34 years. High school education was the most frequent level of education (43.9%) in these individuals. The majority were urban residents (58.9%) and 41.1% lived in rural areas. In this group, similar to the suicide attempt group, married people were the majority (60.4%). Analysis of psychiatric disorders among people with aggressive behaviors also showed that 9% had GAD, 27.6% had MDD, 2.6% had dysthymia, 10.4% had OCD, 7.6% had a history of substance abuse, and 2.8% had a history of alcohol drinking.

 In this study, 39 people had a co-occurring history of suicide attempt and aggressive behavior in the past 12 months, of whom 25 (64.1%) were female and 14 (35.9%) were male. In terms of age group, 28.2% were in the group of 15 to 19 years, 35.9% in the group of 20 to 24 years, 17.9% in the group of 25 to 29 years, and 17.9% in the group of 30 to 34 years. The majority (64.1%) were urban residents and 35.9% lived in rural areas. Also, in terms of history of mental disorders, 17.9% had history of GAD, 71.8% had history of MDD, 2.6% had history of dysthymia, 25.6% had history of OCD, and 7.7% had history of substance abuse. In comparison, when we examined the total sample, it was found the prevalence rates of 4.6% for GAD, 21.6% for MDD, 1.9% for dysthymia, 7.6% for OCD, 5.1% for any substance use disorder, and 1.9% for alcohol drinking.

 As shown in Table 2, the variables of age and education were significant predictors of suicide attempt in the past 12 months. Among the mental disorders, only MDD was a significant predictor of suicide attempt in the past 12 months, but the variables of gender, place of residence, GAD, OCD, dysthymia, and substance use disorder did not play a significant role in predicting suicide attempt in life time.

**Table 2:** Multinomial Logistic Regression

**Variables **		**Suicide (Past 12 Month)**		**Aggression **		**Concurrent**	
		**OR (95% CI)**	***P***	**OR (95% CI)**	***P***	**OR (95% CI)**	***P***
Gender	female	0.78 (0.38-1.66)	0.546	0.98 (0.83-1.15)	0.785	0.87 (0.40-1.91)	0.731
	male	1	-	1	-	1	-
Age	15-19	0.18 (0.6-0.54)	0.002	0.38 (0.28-0.16)	0.001	0.37 (0.18-0.77)	0.008
	20-24	0.25 (0.10-0.65)	0.004	0.74 (0.59-0.93)	0.010	0.40 (0.23-0.68)	0.001
	25-29	0.48 (0.19-1.23)	0.127	0.93 (0.76-1.12)	0.923	0.66 (0.22-1.93)	0.444
	30-34	1	-	1	-	1	-
Education	Illiterate	-	-	0.65 (0.19-2.18)	0.487	-	-
	Primary school	0.31 (0.09-1.12)	0.073	0.63 (0.48-0.82)	0.001	0.24 (0.06-1)	0.050
	Middle school	0.23 (0.08-0.72)	0.012	0.76 (0.59-0.97)	0.026	0.21 (0.17-0.74)	0.015
	High school	0.43 (0.15-1.22)	0.112	0.71 (0.58-0.87)	0.001	0.39 (0.12-1.22)	0.104
	University	1	-	1	0	1	-
Residence	Urban	0.26 (0.38-1.43)	0.378	0.80 (0.68-0.94)	0.801	0.68 (0.33-1.38)	0.286
	Rural	1	-	1	-	1	-
Marital status	Never married	1.93 (0.62-6.02)	0.258	1.53 (0.97-2.40)	0.066	1.13 (0.28-4.50)	0.859
	Married	2.47 (0.84-7.27)	0.100	0.81 (0.53-1.26)	0.358	1.58 (0.41-5.98)	0.504
	Previously married	1	-	1	-	1	-
Psychiatric disorder	GAD	1.07 (0.44-2.58)	0.884	1.84 (1.31-2.59)	0.001	1.30 (0.53-3.18)	0.57
	MDD	7.51 (3.78-14.90)	0.001	1.68 (1.38-2.04)	0.001	8.34 (3.91-17.78)	0.001
	Dysthymia	1.67 (0.20-13.75)	0.633	2.16 (1.21-3.84)	0.009	2.15 (0.25-18.68)	0.57
	OCD	1.59 (0.73-3.48)	0.246	1.68 (1.24-2.28)	0.001	1.81 (0.81-4.04)	0.149
	Substance use disorder	0.95 (0.26-3.45)	0.943	3.92 (2.32-6.64)	0.001	1.02 (0.27-3.83)	0.971

GAD, Generalized anxiety disorder; MDD, Major depressive disorder; OCD, Obsessive-compulsive disorder.

 The results also showed that the variables of age and education, and disorders of GAD (OR = 1.84, *P* = 0.001), MDD (OR = 1.68, *P* = 0.001), OCD (OR = 1.84, *P* = 0.001), dysthymia (OR = 2.16, *P* = 0.009), and substance use disorder (OR = 3.92, *P* = 0.001) were significant predictors of aggression, while the variables of gender, place of residence, and marital status did not play a significant role in predicting aggression. According to the values of OR, it can be said that mental disorders are associated with the occurrence of aggression. Substance use is also associated with aggression.

 As shown in Table 2, the variables of age and education played a significant role in predicting the concurrence of suicide attempt and aggression in the past 12 months. Also, among the mental disorders, MDD (OR = 8.34, *P* = 0.001) predicted the concurrence of suicide attempt and aggression. However, the variables of dysthymia, substance use disorder, GAD and OCD did not play a significant role in predicting the concurrence of suicide attempt and aggression.

## Discussion

 This study aimed to investigate the prevalence of suicide attempt, aggression and the co-occurrence of these two behaviors among a sample of Iranian youth. The results showed that the prevalence of suicide attempt (in life time) in the sample was 5.21%, the prevalence of suicide attempt (in the past 12 months) was 1.50%, the prevalence of aggression was 48.11% and the co-occurrence of suicide attempt and aggression in the study sample was 1.30%. The results also showed that age was involved in predicting suicide attempt. History of suicide attempt was higher among those aged 20 to 24 years compared to the other age groups. The results of our study did not show a significant difference in suicide attempts between men and women, which is inconsistent with some previous studies; for example, a study in a similar population conducted in the Kurdish people of the Ilam province (western Iran) showed that the rate of suicide attempt was higher in women than men.^14^ The results of a review study also showed that suicide attempts are more frequent in women than men.^26^

 Another finding of this study was that that MDD was involved in predicting suicide attempt, meaning that people with MDD were more likely to attempt suicide. Many studies have examined the association between depression and anxiety and suicide. For example, one study found that 84% of people who attempted suicide had symptoms of mental disorders, and 68.3% had symptoms of depression.^12^ The results of another study showed that the prevalence of depressive and anxiety disorders was high in people who attempted suicide.^27^ The results of a longitudinal study showed that depression was a significant risk factor for suicide.^28^ Research has shown that anxiety is a risk factor for suicide and can predict the risk of suicide.^29^ Therefore, in terms of the relationship between depression and anxiety and suicidality, the results of our research are consistent with previous studies.

 In another part of the present study, it was shown that OCD did not play a role in predicting suicide attempt (in the past 12 months). Contrary to this finding, the results of other studies have shown that suicide attempt is a common behavior in people with OCD.^30,31^ The results of another study showed that OCD is a major risk factor for developing suicidal behaviors.^32^ According to the literature, the results of the present study on the relationship between OCD and suicide attempt is inconsistent with previous studies.

 The results of our study showed that substance and alcohol use did not play a significant role in predicting suicide attempt. In this regard, the number of inconsistent studies is significant. For example, the results of a study showed that substance use disorder can raise the risk of suicide.^33^ The results of a review study also showed that suicidal behavior is a major problem among addicts,^34^ and another study showed that substance use disorder can increase the likelihood of suicide.^35^ Previous studies also suggested an association between alcohol drinking and suicide attempt.^36-39^ The results of the present study on the relationship between suicide attempt and substance abuse and alcohol drinking can be a challenging finding that should be considered in future research in the same community.

 Furthermore, we found that GAD, MDD, and dysthymia play a role in predicting aggression, meaning that people with GAD, MDD, and dysthymia are more likely to engage in aggressive behaviors. Reviewing the literature, we find that our results are consistent with previous research on the relationship between anxiety and aggression; for example, the results of a cross-sectional study showed that there is a relationship between high levels of anxiety and aggressive behaviors,^11^ and another confirmed an association between anxiety and aggression.^40^ However, a review of the literature showed that there are inconsistent results in the relationship between depression and aggression. For example, the results of a study showed that people with MDD tended to engage in aggressive behaviors,^10^ and in another study, depressed individuals obtained high scores on the aggression scale.^41^ In contrast, the results of a study showed that there was no significant relationship between depression and aggression.^42^

 The results of this study also showed that OCD was involved in predicting aggression, meaning that aggression was more prevalent in people with OCD. A review of previous studies has concluded that similar to depression, the results in this area also are contradictory; for instance, a study showed an association between OCD and aggression,^43^ but some other studies have not confirmed the relationship between OCD and aggression,^44,45^ inconsistent with the results of our study. These inconsistent results are probably due to age differences in the samples.

 Another important part of the results of this study showed that substance use disorder can predict the occurrence of aggressive behaviors. Similarly, some previous studies have shown that substance use disorder is associated with an increase in aggression,^46^ and other studies have shown an association between substance use disorder and aggression.^47,48^ Our study also showed that alcohol drinking did not play a role in predicting aggression. This finding is inconsistent with previous research because they showed that there was a relationship between alcohol drinking and aggression.^46,49^

 The results of the present study showed that MDD was the most important risk factor for the co-occurrence of aggression and suicide attempt in young people, which means that the prevalence of this mental disorders was higher in people who had aggression and attempted suicide at the same time. However, the role of GAD, OCD, dysthymia, substance use disorder and alcohol drinking in this regard was not significant. In a study aimed at investigating the correlations between co-occurrence of aggression and suicide in veterans, the results showed that alcohol drinking plays a significant role in co-occurrence of aggression and suicide,^19^ which is inconsistent with the findings of the present study. The results of the present study also showed that age and education played a significant role in predicting the co-occurrence of suicide attempt and aggression, but there was no significant difference observed between women and men. However, in a previous study, the co-occurrence of aggression and suicide attempt in women was higher than men.^19^

 This study was conducted on a sample of young people in western Iran, so more research is suggested to better understand the nature of aggressive behaviors and suicide attempts among young people. There is also a need for research in other societies and other ages. In this study, we encounter several other limitations, including the fact that very few of the participants had co-occurrence of suicide attempt and aggression, and this could lead to bias in the results of regression analysis. The number of items in the subgroups may lead to scattered data bias, which makes it difficult to interpret logistic regression results.^50^ Also in this study, we did not examine personality disorders which can play a significant role in aggression and suicide, and this is a point that should be considered.

 In conclusion, the present study showed that the demographic variables of age and education play a role in predicting the co-occurrence of suicide attempt and aggression in young people in western Iran. Also, among the mental disorders, MDD was involved in predicting the concurrence of suicide attempt and aggression. Therefore, in order to reduce the attempted suicide and aggression in young people, it is recommended to adopt targeted and planned measures to treat and prevent mental disorders.
